# Supplementary material for: Progenitors oppositely polarize WNT activators and inhibitors to orchestrate tissue development
Source: eLife. 2020 Apr 20;9:e54304. doi: 10.7554/eLife.54304 (PMC7224699; doi:10.7554/eLife.54304)
Supplement: Figure 2—figure supplement 2—source data 1. [file elife-54304-fig2-figsupp2-data1.docx]

**Table 1**. Cell adhesion transcripts upregulated in *Apc null* WNThi cells (Geneontology – PANTHER Classification System)

| Log2  Fold Change  ≥1.5 | *Gpc4 Cdh22 Lamc3 Fblim1 Pcdh10 Atrnl1 Cdh6 Ptprd Dsg4 Sdk2 Plxna4 Ntng2 Tnfsf9 Ccdc141 Pcdh19 Cdh11 Ctnnd2 Cnr1 Pvrl3 Parvg Ntng1 Parvb Gpc6* |
| --- | --- |

Matos et al, Figure 2 – figure supplement 2 – source data 1
