# Supplementary material for: Progenitors oppositely polarize WNT activators and inhibitors to orchestrate tissue development
Source: eLife. 2020 Apr 20;9:e54304. doi: 10.7554/eLife.54304 (PMC7224699; doi:10.7554/eLife.54304)
Supplement: Figure 3—figure supplement 2—source data 1. — Shown is a list of transcripts that are shared between the Apc null WNThi signature and the wild-type WNThi signature. [file elife-54304-fig3-figsupp2-data1.docx]

Table 2. Hair follicle development WNT^hi^ signature genes

|  | Overlapping transcripts of *Apc null* WNT^hi^ signature and WT WNT^hi^ signature |
| --- | --- |
| Log2  Fold Change  ≥1.5 | *Lhx2, Pdzrn3, Shh, Frem1, Tgfb2, Wif1, Notum, Bmp4, Scube1, Slc40a1, Nrp2, 3632451O06Rik, Bach2, Cnr1, Mgp, Cyfip2, Crym, Sox13, Nptx2 Basp1, Sp5, Ltbp1, Gad1, Adamts17, Zbtb10, Cldn6, Pde4d, Id4, Tll1, Syt5, Tbx1, Nkd1, Fam134b, Ptprv, Clip3, Ism1, Lbh, Stap1, Gng8, Dach1, Abcc4, Rem2, Rnf32, Pdgfrl, Mfap2, Csrp2, Irs2, A430010J10Rik, Spo11, Cmtm8, Hmcn1, Man1a, Slc14a1, Pcdh19, Uchl1, Smad7, Krt23, Zdhhc14, H2-K1, Csgalnact1, Runx2, Casz1, Gm17441, Tmem151b, Runx1, Sox21, Grik5, Plk3, Dusp6, 4732444A12Rik,Gm11768, Fads2, Nme5, Gxylt2, Six4, Krt18, Cpe, Khdrbs3, A930001N09Rik, Msx2, Robo1, Pvrl3, Amot, Chst11, Setbp1, Ier5l, Tnfsf9, Ism2, Mylip, Fermt2, Sh3gl3, Greb1, Bmp2, Gadd45g, Ptch2, Thsd4, Ank, Padi3, Clcf1, Rasgef1b, Dennd2a, Nampt, Bai2, Edar, Bambi, Cdc42ep3, Fxyd6, Srgap1, Gm20544, Acsl3,Utp14b, Krt33a, Krt34, Wnt10b, Glul, Id2, Flrt2, 9930012K11Rik, Rhob, Rgs9,Shb, Ajap1, Spry4, Serinc5, Pkdcc, Smad6, Gpm6b, Lrp4, Atg16l2, Hebp1, Adamtsl2, Sel1l3, Hs3st3b1, Rai14, Cadm4, Traf4, Lgr6, Orai2, Krt8, Trib2, Fam43a* |

Matos et al, Figure 3 – figure supplement 2 – source data 1
