# Supplementary material for: Progenitors oppositely polarize WNT activators and inhibitors to orchestrate tissue development
Source: eLife. 2020 Apr 20;9:e54304. doi: 10.7554/eLife.54304 (PMC7224699; doi:10.7554/eLife.54304)
Supplement: Supplementary file 1. [file elife-54304-supp1.docx]

| **Key Resources Table** | | | | |
| --- | --- | --- | --- | --- |
| **Reagent type**  **(species) or**  **resource** | **Designation** | **Source or Reference** | **Identifiers** | **Additional information** |
| experimental model, mouse strain | Mouse*: Apcfl/fl* | (Kuraguchi et al. 2006) | Kucherlapati Lab |  |
| experimental model, mouse strain | Mouse*: Axin2-LacZ* | The Jackson Laboratory;  (Lustig et al. 2002) |  |  |
| experimental model, mouse strain | Mouse: CD1/ICR | Charles River | Fuchs Lab |  |
| experimental model, mouse strain | Mouse: Fucci | *595, Riken*  (Sakaue-Sawano et al. 2008) |  |  |
| experimental model, mouse strain | Mouse: *Krt14-rtTA* | (Nguyen, Rendl, and Fuchs 2006) | Fuchs Lab |  |
| experimental model, mouse strain | Mouse: *Lhx2-EGFP* | The Gene Expression Nervous System Atlas (GENSAT) Project, NINDS Contracts N01NS02331 & HHSN271200723701C to The Rockefeller University, New York, NY, USA |  |  |
| experimental model, mouse strain | Mouse*: Notum KO* and  *Notum fl/fl* | (Canal et al. 2016) | Jean Paul-Vincent Lab |  |
| experimental model, mouse strain | Mouse*: Rosa26Flox-Stop-Flox-tdTom* | The Jackson Laboratory |  |  |
| experimental model, mouse strain | Mouse: *Wif1-KO* | Igor Dawid (NIH) |  |  |
| antibody | anti-human APC  (rabbit polyclonal) | Sigma-Aldrich | Cat#:  HPA013349  RRID:  AB_1844913 | IHC  (1:500) |
| antibody | anti-human, canine, bovine, chicken β-catenin (15B8)  (mouse monoclonal) | Sigma-Aldrich | Cat#:  C7207  RRID:  AB_476865 | IHC  (1:1000) |

| antibody | anti-mouse β-catenin  (mouse monoclonal) | BD Biosciences | Cat#:  610154  RRID:  AB_397555 | IF  (1:200) |
| --- | --- | --- | --- | --- |
| antibody | anti-human and mouse CD49f-PE/Cy7 (Integrin-α6)  (rat monoclonal) | BioLegend | Cat#:  313622  RRID:  AB_2561705 | FACS  (1:200) |
| antibody | anti-mouse CD31-APC  (rat monoclonal) | BioLegend | Cat#:  102410  RRID:  AB_312905 | FACS (1:200) |
| antibody | anti-mouse CD140a-APC  (rat monoclonal) | BioLegend | Cat#:  135908  RRID:  AB_2043970 | FACS (1:200) |
| antibody | anti-human, mouse E-Cadherin (24E10)  (rabbit monoclonal) | Cell Signaling | Cat#:  3195S  RRID:  AB_2291471 | IF  (1:500) |
| antibody | anti-human, mouse FZD10  (rabbit polyclonal) | MyBioSource | Cat#:  MBS9606335 | IF  (1:200) |
| antibody | all species anti-GFP/YFP  (chicken polyclonal) | Abcam | Cat#:  Ab13970  RRID:  AB_300798 | IF  (1:2000) |
| antibody | anti-human, mouse, rat, monkey, Phospho-Histone H2A.X (Ser139) (20E3)  (rabbit monoclonal) | Cell Signaling | Cat#:  9718S  RRID:  AB_2118009 | IF  (1:200) |
| antibody | anti-mouse Integrin-β4 (346-11A) (CD104)  (rat monoclonal) | BD Biosciences | Cat#:  553745  RRID:  AB_395027 | IF  (1:500) |
| antibody | anti-human, mouse, rat LEF1 (C12A5)  (rabbit monoclonal) | Cell Signaling | Cat#:  2230  RRID:  AB_823558 | IF  (1:300) |
| antibody | anti-mouse LEF1  (rabbit polyclonal) | Fuchs Lab |  | IF  (1:300) |
| antibody | anti-mouse LEF1  (guinea pig polyclonal) | Fuchs Lab |  | IF  (1:500) |
| antibody | anti-mouse LHX2  (rabbit polyclonal) | Fuchs Lab |  | IF  (1:2000) |
| antibody | all species anti-MYC (71D10)  (rabbit monoclonal) | Cell Signaling | Cat#:  2278  RRID:  AB_490778 | IF  (1:500) |

| antibody | anti-human NOTUM  (rabbit polyclonal) | Sigma-Aldrich | Cat#  HPA023041 RRID: AB_1854569 | IF  (1:100) |
| --- | --- | --- | --- | --- |
| antibody | anti-mouse P-CADHERIN  (goat polyclonal) | R&D | Cat#:  AF761  RRID:  AB_355581 | IF  (1:300) |
| antibody | all species anti-RFP (5F8)  (rat monoclonal) | Chromotek | Cat#:  5f8-100  RRID:  AB_2336064 | IF  (1:1000) |
| antibody | anti-human, mouse, rat Phospho-Smad1 (Ser463/465)/ Smad5 (Ser463/465)/ Smad9 (Ser465/467) (D5B10)  (rabbit monoclonal) | Cell signaling | Cat#:  13820  RRID:  AB_2493181 | IF  (1:200) |
| antibody | anti-mouse SHH  (goat polyclonal) | R&D | Cat#:  AF445 | IF  (1:50) |
| antibody | anti-human SOX2 (EPR3131)  (rabbit monoclonal) | Abcam | Cat#:  ab92494  RRID:  AB_10585428 | IF  (1:200) |
| antibody | anti-mouse SOX9  (guinea pig polyclonal) | Fuchs Lab |  | IF  (1:1000) |
| antibody | anti-human, mouse TCF1/TCF7 (C63D9)  (rabbit monoclonal) | Cell signaling | Cat#:  2203 | IF  (1:200) |
| antibody | anti-mouse, rat, chinese hamster TGN46  (rabbit polyclonal) | Abcam | Cat#:  ab16059  RRID:  AB_443307 | IF  (1:200) |
| antibody | anti-mouse WIF1  (goat polyclonal) | R&D | Cat#:  AF135  RRID:  AB_354748 | IF  (1:100) |
| antibody | anti-chicken AF488 conjugated antibody  (donkey polyclonal) | Jackson ImmunoResearch | Cat#:  703-545-155  RRID:  AB_2340375 | IF  (1:1000) |
| antibody | anti-goat AF488 conjugated antibody  (donkey polyclonal) | Jackson ImmunoResearch | Cat#:  705-545-003  RRID:  AB_2340428 | IF  (1:1000) |
| antibody | anti-goat RRX conjugated antibody  (donkey polyclonal) | Jackson ImmunoResearch | Cat#:  705-295-147  RRID:  AB_2340423 | IF  (1:1000) |

| antibody | anti-goat AF647 conjugated antibody  (donkey polyclonal) | Jackson ImmunoResearch | Cat#:  705-605-003  RRID:  AB_2340436 | IF  (1:1000) |
| --- | --- | --- | --- | --- |
| antibody | anti-guinea pig AF488 conjugated antibody  (donkey polyclonal) | Jackson ImmunoResearch | Cat#:  706-545-148  RRID:  AB_2340472 | IF  (1:1000) |
| antibody | anti-guinea pig RRX conjugated antibody  (donkey polyclonal) | Jackson ImmunoResearch | Cat#:  706-295-148  RRID:  AB_2340468 | IF  (1:1000) |
| antibody | anti-guinea pig AF647 conjugated antibody  (donkey polyclonal) | Jackson ImmunoResearch | Cat#:  706-605-148  RRID:  AB_2340476 | IF  (1:1000) |
| antibody | anti-rabbit AF488 conjugated secondary  (donkey polyclonal) | Jackson ImmunoResearch | Cat#:  711-545-152  RRID:  AB_2313584 | IF  (1:1000) |
| antibody | anti-rabbit RRX conjugated secondary  (donkey polyclonal) | Jackson ImmunoResearch | Cat#:  711-295-152  RRID:  AB_2340613 | IF  (1:1000) |
| antibody | anti-rabbit AF647 conjugated secondary  (donkey polyclonal) | Jackson ImmunoResearch | Cat#:  711-605-152  RRID:  AB_2492288 | IF  (1:1000) |
| antibody | anti-rat AF488 conjugated antibody  (donkey polyclonal) | Jackson ImmunoResearch | Cat#:  712-546-153  RRID:  AB_2340686 | IF  (1:1000) |
| antibody | anti-rat RRX conjugated antibody  (donkey polyclonal) | Jackson ImmunoReserach | Cat#:  712-295-153  RRID:  AB_2340676 | IF  (1:1000) |
| antibody | anti-rat AF647 conjugated antibody  (donkey polyclonal) | Jackson ImmunoResearch | Cat#:  712-605-153  RRID:  AB_2340694 | IF  (1:1000) |
| sequenced-based reagent | pCR4-*mApcdd1* | Angela Cristiano  Lab |  |  |
| sequenced-based reagent | pGEMT-*mDkk4* | David Schlessinger Lab |  |  |
| sequenced-based reagent | pCRII-*mWif1_3* | Fuchs Lab |  |  |
| sequenced-based reagent | PCRII-*Wnt10b* | Fuchs Lab |  |  |

| recombinant DNA reagent | *pLKO.1-Pgk-Cre-mRFP*  (plasmid) | Fuchs Lab |  |  |
| --- | --- | --- | --- | --- |
| recombinant DNA reagent | *pLKO.1-Pgk-Cre-EGFP*  (plasmid) | This paper |  | *See Materials and Methods section: mouse strains, lentiviral transduction and constructs* |
| recombinant DNA reagent | *pLKO-TK-12xTOP-EGFP-Pgk-Cre*  (plasmid) | This paper |  | *See Materials and Methods section: mouse strains, lentiviral transduction and constructs* |
| recombinant DNA reagent | *LV-TRE-Gene-Pgk-H2BGFP*  (plasmid) | Fuchs Lab |  |  |
| recombinant DNA reagent | *pGEM-Apcdd-Myctag*  (plasmid) | Origene | Cat#:  MR225129 |  |
| recombinant DNA reagent | *pGEM-mBmp4*  (plasmid) | Sino Biological Inc. | Cat#:  MG50439-G |  |
| recombinant DNA reagent | *pCMV6-Dkk4-Myctag*  (plasmid) | Origene | Cat#:  MR202533 |  |
| recombinant DNA reagent | *pCMV6-Notum-Myctag* (plasmid) | Origene | Cat#:  MR217230 |  |
| recombinant DNA reagent | *pCMV6-Wif1-Myctag*  (plasmid) | Origene | Cat#:  MR202510 |  |
| recombinant DNA reagent | *pCMV6-Wnt3-Myctag*  (plasmid) | Origene | Cat#:  MR222492 |  |
| recombinant DNA reagent | *pCMV6-Wnt10b-Myctag*  (plasmid) | Origene | Cat#:  MR224739 |  |
| recombinant DNA reagent | *LV-TRE-Apcdd-M-Pgk-H2BGFP*  (plasmid) | This paper |  | *See Materials and Methods section: mouse strains, lentiviral transduction and constructs* |
| recombinant DNA reagent | *LV-TRE-Bmp4-Pgk-H2BGFP*  (plasmid) | Fuchs Lab |  |  |
| recombinant DNA reagent | *LV-TRE-Dkk4-M-Pgk-H2BGFP*  (plasmid) | This paper |  | *See Materials and Methods section: mouse strains, lentiviral transduction and constructs* |
| recombinant DNA reagent | *LV-TRE-Notum-M-Pgk-H2BGFP*  (plasmid) | This paper |  | *See Materials and Methods section: mouse strains, lentiviral transduction and constructs* |

| recombinant DNA reagent | *LV-TRE-Notum-Aqp4-M-Pgk-H2BGFP*  (plasmid) | This paper |  | *See Materials and Methods section: mouse strains, lentiviral transduction and constructs* |
| --- | --- | --- | --- | --- |
| recombinant DNA reagent | *LV-TRE-Wif1-M-Pgk-H2BGFP*  (plasmid) | This paper |  | *See Materials and Methods section: mouse strains, lentiviral transduction and constructs* |
| recombinant DNA reagent | *LV-TRE-Wif1-Aqp4-M-Pgk-H2BGFP*  (plasmid) | This paper |  | *See Materials and Methods section: mouse strains, lentiviral transduction and constructs* |
| recombinant DNA reagent | *LV-TRE-Wnt3-M-Pgk-H2BGFP*  (plasmid) | This paper |  | *See Materials and Methods section: mouse strains, lentiviral transduction and constructs* |
| recombinant DNA reagent | *LV-TRE-Wnt10b-M-Pgk-H2BGFP*  (plasmid) | This paper |  | *See Materials and Methods section: mouse strains, lentiviral transduction and constructs* |
| chemical compound, drug | Doxycycline | Sigma | Cat#:  D9891 |  |
| chemical compound, drug | LGK974 | Cayman Chemical | Cat#:  14072 |  |
| chemical compound, drug | Tunicamycin | Milipore Sigma | Cat#:  504570 |  |
| chemical compound, drug | 16% Paraformaldehyde Solution | Electron Microscopy Science | Cat#:  15700 |  |
| commercial assay or kit | Click-it Edu Alexa Fluor 647 Imaging kit | Life Technologies | Cat#:  C10340 |  |
| commercial assay or kit | Direct-zol RNA MiniPrep kit | Zymo Research | Cat#:  R2050 |  |
| commercial assay or kit | ImmPRESS® Universal PLUS Polymer Kit, Peroxidase (Horse Anti-Mouse/Rabbit IgG) | Vector Laboratories | Cat#:  MP-7800 |  |
| commercial assay or kit | ImmPACT® DAB Peroxidase (HRP) Substrate | Vector Laboratories | Cat#:  SK-4105 |  |
| commercial assay or kit | M.O.M.® (Mouse on Mouse) Basic Kit | Vector Laboratories | Cat#:  BMK-2202 |  |

| software, algorithm | Adobe Illustrator CS5 | Adobe.com | RRID:  SCR_010279 |  |
| --- | --- | --- | --- | --- |
| software, algorithm | FACS DiVa software | BD Biosciences | RRID:  SCR_001456 |  |
| software, algorithm | Fiji (Image J) | (<https://fiji.sc/>) | RRID:  SCR_003070 |  |
| software, algorithm | FlowJo Software | BD Biosciences | RRID:  SCR_008520 |  |
| software, algorithm | Graphpad Prism 8 | Graphpad.com | RRID:  SCR_002798 |  |
| software, algorithm | Gene Set Enrichment Analysis (GSEA) | (Subramanian et al. 2005) | RRID:  SCR_003199 |  |
| software, algorithm | PANTHER Classification System | GENEONTOLOGY | RRID:  SCR_015893 |  |
| software, algorithm | R studio | Rstudio.com | RRID:  SCR_000432 |  |
| other | SlowFade™ Diamond Antifade Mountant (Invitrogen) | TermoFisher | Cat#:  S36963 |  |
| other | Dispase | Gibco | Cat#:  17105-041 |  |
| other | Trypsin-EDTA 0.25% | Gibco | Cat#:  25200056 |  |
| other | Trizol LS  (Invitrogen) | TermoFisher | Cat#:  10296010 |  |
